# An Active C-Terminally Truncated Form of Ca^**2+**^/Calmodulin-Dependent Protein Kinase Phosphatase-N (CaMKP-N/PPM1E)

**DOI:** 10.1155/2013/134813

**Published:** 2013-08-07

**Authors:** Atsuhiko Ishida, Kumiko Tsumura, Megu Oue, Yasuhiro Takenaka, Yasushi Shigeri, Naoki Goshima, Yasuhiro Ishihara, Tetsuo Hirano, Hiromi Baba, Noriyuki Sueyoshi, Isamu Kameshita, Takeshi Yamazaki

**Affiliations:** ^1^Laboratory of Molecular Brain Science, Graduate School of Integrated Arts and Sciences, Hiroshima University, Higashi-Hiroshima 739-8521, Japan; ^2^National Institute of Advanced Industrial Science and Technology, Ikeda, Osaka 563-8577, Japan; ^3^Department of Life Sciences, Faculty of Agriculture, Kagawa University, Kagawa 761-0795, Japan

## Abstract

Ca^2+^/calmodulin-dependent protein kinase phosphatase (CaMKP/PPM1F) and its nuclear homolog CaMKP-N (PPM1E) are Ser/Thr protein phosphatases that belong to the PPM family. CaMKP-N is expressed in the brain and undergoes proteolytic processing to yield a C-terminally truncated form. The physiological significance of this processing, however, is not fully understood. Using a wheat-embryo cell-free protein expression system, we prepared human CaMKP-N (hCaMKP-N(WT)) and the truncated form, hCaMKP-N(1–559), to compare their enzymatic properties using a phosphopeptide substrate. The hCaMKP-N(1–559) exhibited a much higher *V*_max_ value than the hCaMKP-N(WT) did, suggesting that the processing may be a regulatory mechanism to generate a more active species. The active form, hCaMKP-N(1–559), showed Mn^2+^ or Mg^2+^-dependent phosphatase activity with a strong preference for phospho-Thr residues and was severely inhibited by NaF, but not by okadaic acid, calyculin A, or 1-amino-8-naphthol-2,4-disulfonic acid, a specific inhibitor of CaMKP. It could bind to postsynaptic density and dephosphorylate the autophosphorylated Ca^2+^/calmodulin-dependent protein kinase II. Furthermore, it was inactivated by H_2_O_2_ treatment, and the inactivation was completely reversed by treatment with DTT, implying that this process is reversibly regulated by oxidation/reduction. The truncated CaMKP-N may play an important physiological role in neuronal cells.

## 1. Introduction

**Figure 1:**
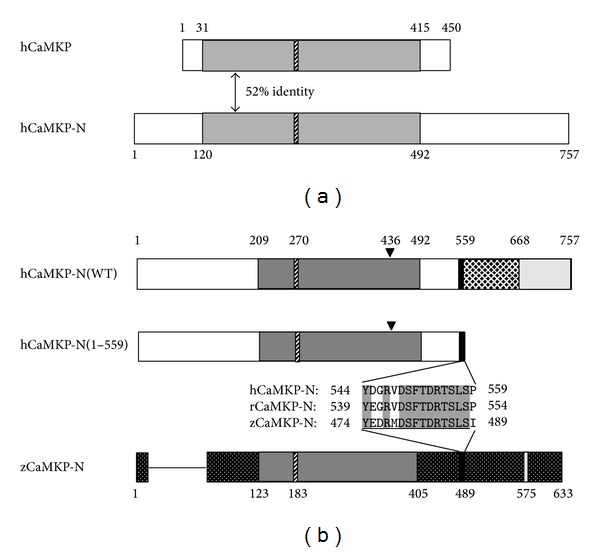
Schematic representation of hCaMKP, hCaMKP-N(WT), hCaMKP-N(1–559), and zCaMKP-N. (a) Comparison of the primary structure of hCaMKP-N with that of hCaMKP. The regions that show significant homology are shown in grey. (b) Domain structures of hCaMKP-N(WT), hCaMKP-N(1–559), and z-CaMKP-N. Catalytic domains are shown in dark grey, and the hatched bars within them indicate the PP2C motif that is characteristic of PPM family phosphatases. Black bars show the antibody recognition site. In hCaMKP-N, the recognition site is located just to the N-terminal side of the putative processing site. The amino acid sequences of the antibody recognition site in human, rat, and zebrafish CaMKP-N are also indicated. Underline shows the amino acid sequence used for generating the anti-CaMKP-N antibody. Closed arrowheads show the position of Cys436, which may be responsible for redox regulation of the phosphatase activity (see text). It has been reported that two nuclear localization signals are located in the region that is C-terminal side to Arg668 on hCaMKP-N (shown in light grey). The 575–587 region of zCaMKP-N reportedly functions as a nuclear localization signal (shown in light grey) [8]. The amino acid residue numbers of the respective phosphatases are also shown.

Ca^2+^/calmodulin-dependent protein kinase phosphatase (CaMKP/PPM1F/POPX2) was first identified in rat brain as a unique protein phosphatase that specifically dephosphorylates and regulates multifunctional CaMKs, including CaMKI, II, and IV [1–4]. Thereafter, another protein phosphatase with 52% identity in the catalytic domain to human CaMKP was found in the human cDNA databases (Figure 1(a)). When the cDNA was expressed in COS cells, the phosphatase encoded by the cDNA was localized to the nucleus, in contrast to CaMKP, which was exclusively found in the cytosol. Therefore, we named the enzyme CaMKP-N for its localization in the nucleus [5]. Subsequently, other groups named this enzyme POPX1 [6] or PPM1E [7] and reported that it is involved in the negative regulation of the p21-activated protein kinase [6] and the 5′-AMP-activated protein kinase [7]. Gene knockdown experiments for zebrafish CaMKP-N (zCaMKP-N) using morpholino-based antisense oligonucleotides indicated that CaMKP-N is essential for the early development of the brain and spinal cord in zebrafish [8]. 

 We also showed that the proteolytic processing of zCaMKP-N plays a critical role in the regulation of its catalytic activity, subcellular localization, and substrate targeting [9]. In accordance with these results, Kitani et al. [10] suggested that the majority of CaMKP-N undergoes proteolytic processing to generate a 90 kDa fragment in rat brain that is localized to the cytosol. Since the amino acid sequence homology between zCaMKP-N and human CaMKP-N (hCaMKP-N) is only 48% and the molecular size of zCaMKP-N is much smaller than the size of hCaMKP-N (by more than 100 amino acid residues, Figure 1(b)), it is difficult to predict, on the basis of sequence homology, whether the 90 kDa fragment is a mammalian counterpart for the active fragment of zCaMKP-N that is generated by proteolytic processing in zebrafish. It is clinically significant to explore the enzymatic properties of hCaMKP-N, which may provide a molecular basis for drug development because CaMKP-N has been suggested to regulate the 5′-AMP-activated protein kinase involved in the pathogenesis of diabetes [7]. Unfortunately, difficulty in preparing hCaMKP-N in a sufficient purity and quantity has hampered the biochemical characterization of this enzyme. Our initial attempt to obtain pure full-length hCaMKP-N using transfected Sf9 cells failed due to proteolysis during the expression and purification. We performed only a preliminary characterization of hCaMKP-N using a mixed preparation that contained both full-length hCaMKP-N and its truncated fragment, which was generated during the preparation [5]. Therefore, no information has been available about the enzymatic properties and the physiological importance of each species of hCaMKP-N. 

 In this study, we utilized a wheat-embryo cell-free protein expression system to separately prepare full-length hCaMKP-N and hCaMKP-N(1–559), a human counterpart to the 90 kDa truncated fragment found in the rat brain. Using these preparations, we compared the kinetic properties of full-length hCaMKP-N and the truncated fragment, and we found that the truncated fragment had much higher phosphatase activity than the full-length form. We also examined the enzymatic properties of the truncated hCaMKP-N, which had not been characterized in detail, and discussed the physiological importance of the processing. 

## 2. Materials and Methods

### 2.1. Materials

The phosphopeptides, pp2 (MHRQE**T(p)**VDC), pp4 (MHRQE**S(p)**VDC), pp6 (MHRQE**Y(p)**VDC), and pp10 (YGGMHRQE**T(p)**VDC), were synthesized using a Shimadzu PSSM-8 automated peptide synthesizer and purified by reverse-phase HPLC on a C_18_ column [5, 11]. The identity and purity of the peptides were confirmed by time-of-flight mass spectrometry. The postsynaptic density (PSD) fraction was purified from rat cerebral cortex as described by Sahyoun et al. [12]. The anti-CaMKP-N antibody was obtained as previously described [8]. Monoclonal anti-CaMKII*α* (MAb CB*α*-2) was purchased from Life Technologies. The anti-active CaMKII was from Promega. The biotin-conjugated rabbit anti-mouse IgG was obtained from ZYMED. The anti-rabbit Ig, biotinylated species-specific antibody, streptavidin-conjugated horseradish peroxidase, and the Ni^2+^-Sepharose high-performance resin were from GE Healthcare. An enhanced chemiluminescence detection agent, SuperSignal West Femto Maximum Sensitivity Substrate, was from Thermo Scientific. DNase-free RNase was from Boeringer Mannheim. Hydrogen peroxide, Quick CBB, and okadaic acid were purchased from Wako Pure Chemical Industries, and the 1-amino-8-naphthol-2,4-disulfonic acid (ANDS) was from Tokyo Chemical Industry. Calyculin A was from Millipore/Upstate. All other agents were obtained from Nacalai Tesque or Sigma-Aldrich. 

### 2.2. Construction of Plasmids

The pEU-E01-CaMKPN vector, which encodes the wild type (WT) hCaMKP-N with an N-terminal 6x His tag, was prepared as described in the following. The following primers were used for PCR: CaMKPN His-UP1 (5′-GGA TAT CTA TGT CGT ACT ACC ATC ACC ATC ACC ATC ACG CCG GCT GCA TCC CTG AGG AGA A-3′) with an *EcoR*V site (underlined) and CaMKPN LP1 (5′-GGT CGA CTT ATT CTA TTT TAT AGC TCC AAG GAA GAT-3′) with a *Sal*I site (underlined). The PCR was performed in a PTC-200 Thermal Cycler (MJ Research) for 20 cycles, each consisting of denaturation for 5 s at 98°C, annealing for 30 s at 60°C, and extension for 2 min at 68°C, in the presence of 3% (v/v) dimethyl sulfoxide using Pyrobest DNA Polymerase (Takara) and the plasmid DNA containing hCaMKP-N cDNA (AK289966, HuPEX clone FLJ76651) as the template. After gel purification, the amplified product was digested with *EcoR*V and *Sal*I and cloned into pCR4Blunt-TOPO (Invitrogen). The plasmid DNA was then sequenced with the ABI PRISM 3100 Genetic Analyzer (Applied Biosystems) to confirm the DNA sequence of the cloned insert. The plasmid was digested with *EcoR*V and *Sal*I, and the insert purified by gel purification was ligated into pEU-E01-MCS (CellFree Science) at the *EcoR*V and *Sal*I sites to generate pEU-E01-CaMKPN, which encodes the full-length hCaMKP-N with an N-terminal 6× His-tag. The pEU-E01-CaMKPN(1–559) vector, which encodes the C-terminal deletion mutant hCaMKP-N(1–559) with an N-terminal 6× His tag, was prepared using an inverse PCR mutagenesis kit (KOD-Plus-Mutagenesis Kit, TOYOBO) according to the manufacturer's instructions, with pEU-E01-CaMKPN as the template. The PCR was performed in a PC707 Thermal Cycler (ASTEC) for 10 cycles, each consisting of denaturation for 10 s at 98°C, annealing for 30 s at 60°C, and extension for 6 min at 68°C, in the presence of 4.8% (v/v) dimethyl sulfoxide with the sense primer (5′-TAG ATC CCA AAT CAA CGT GCT GGA AGA C-3′, underline shows the site of mutation) and the antisense primer (5′-TGG GCT CAG GCT AGT TCT ATC AGT G-3′). The PCR product was digested with *Dpn*I, and after gel purification, the amplified product was phosphorylated and self-ligated to generate pEU-E01-CaMKPN(1–559). The sequence of the mutated insert was confirmed by DNA sequencing.

### 2.3. The Cell-Free Expression of Recombinant hCaMKP-Ns and Its Purification

Cell-free expression of hCaMKP-N(WT) and hCaMKP-N(1–559) was carried out using a wheat-embryo cell-free protein expression system, the WEPRO 1240H Expression Kit (CellFree Sciences), according to the manufacturer's instruction, with either pEU-E01-CaMKPN or pEU-E01-CaMKPN(1–559). The *in vitro* translation reactions were conducted in a 96-well microtiter plate at 15°C for 18–20 hs. After the translation reaction, DNase-free RNase (24 *μ*g/mL) and the cOmplete Mini EDTA-free protease inhibitor cocktail (Roche) (1 tablet/10 mL) were added to the translation reaction mixture, and the mixture was incubated for 30 min on ice. To 3 mL of the mixture, 0.96 mL of 50% (v/v) suspension of Ni^2+^-Sepharose resin, which had been equilibrated and suspended in 20 mM Tris-HCl (pH 7.5) containing 0.2% (v/v) Tween 40, 10 mM imidazole, 300 mM NaCl, and 1 mM DTT, was added, and the mixture was then gently rocked at 4°C for 1 h. All of the following purification procedures were carried out at 4°C. The gel slurry was poured into an empty column (0.12 × 14 cm), and the flow-through fraction was allowed to drain. The column was washed with 5 mL of 20 mM Tris-HCl (pH 7.5) containing 0.05% (v/v) Tween 40, 20 mM imidazole, 1.5 M NaCl, and 1 mM DTT. For CaMKP-N(WT), the column was further washed with 2.5 mL of 20 mM Tris-HCl (pH 7.5) containing 0.05% (v/v) Tween 40, 100 mM imidazole, 300 mM NaCl, and 1 mM DTT, or for CaMKP-N(1–559), the column was further washed with 20 mM Tris-HCl (pH 7.5) containing 0.05% (v/v) Tween 40, 40 mM imidazole, 300 mM NaCl, and 1 mM DTT. The column was eluted with 2.5 mL of 20 mM Tris-HCl (pH 7.5) containing 0.05% (v/v) Tween 40, 250 mM imidazole, 300 mM NaCl, and 1 mM DTT, and the eluate was concentrated using a centrifugal filter unit (10,000 MWCO, Millipore). Glycerol was added to a final concentration of 30% (v/v). The purified enzymes were aliquoted and could be stored at −80°C for several months without any detectable loss in activity. 

### 2.4. Protein Phosphatase Assays

Protein phosphatase assays using a phosphopeptide as a substrate were performed as previously described [13]. The phosphopeptides used in this study were derived from the amino acid sequence around the Thr286 autophosphorylation site on CaMKII*α* (CaMKII(281–289) = pp2) or its analogs (pp4, pp6, pp10). The reaction was initiated by adding enzyme, and the reaction mixture was incubated at 30°C for 45 min. The amount of inorganic phosphate released in the mixture during the incubation was determined by malachite green assay [11]. The Michaelis-Menten kinetic parameters were determined from a direct fit to the Michaelis-Menten equation using a nonlinear regression program (DeltaGraph, version 6.0, Red Rock Software) as previously described [11]. The tested compounds were added to the reaction systems mentioned above at the indicated concentrations. 

 The protein phosphatase assay using autophosphorylated CaMKII as a phosphoprotein substrate was performed as described [13] with the following modifications. The PSD fraction (905 *μ*g/mL) was incubated at 5°C for 10 min in the reaction mixture (100 *μ*L) containing 40 mM Hepes-NaOH (pH 8.0), 5 mM Mg(CH_3_COO)_2_, 0.1 mM EGTA, 1 *μ*M calmodulin, 0.8 mM CaCl_2_, 0.01% Tween 20, and 50 *μ*M nonradioactive ATP to autophosphorylate the CaMKII found in the PSD fraction. After the reaction, the mixture was immediately diluted with 1 mL of ice-cold wash buffer consisting of 50 mM Tris-HCl (pH 7.5), 0.2 M NaCl, 0.05% Tween 40, 0.1 *μ*M calyculin A, and 0.1 mM DTT, and then it was centrifuged for 10 min at 4°C in a microcentrifuge at maximum speed. The supernatant was removed, and the precipitate was resuspended with the ice-cold wash buffer and centrifuged again as described previously. The washing procedure was repeated five more times. The precipitate obtained was resuspended in 100 *μ*L of the washing buffer and stored at −80°C until it was used. The phosphatase reaction was carried out in a reaction mixture containing 50 mM Tris-HCl (pH 7.5), 2 mM MnCl_2_, 0.1 mM EGTA, and 0.01% Tween 20. Western blotting analysis using anti-active CaMKII (anti-PCaMKII, 1 : 1000 dilution) was performed to estimate the extent of autophosphorylation at the Thr286 site on CaMKII. After the detection of autophosphorylated CaMKII, the blot was treated with Blot Restorte Membrane Rejuvenation Kit (Millipore) according to the manufacturer's instructions, so that it could be reprobed. To confirm the amount of total CaMKII on the blot, the rejuvenated blot was reprobed with a monoclonal anti-CaMKII*α* (CB*α*-2, 1 : 500 dilution). 

### 2.5. Binding of hCaMKP-N(1–559) to PSD

hCaMKP-N(1–559) (1 *μ*g) was incubated with the PSD fraction (1 *μ*g) in 50 mM Tris-HCl (pH 7.5) (10 *μ*L) on ice for 1 h and then centrifuged for 5 min at 4°C in a microcentrifuge at maximum speed. The pellet fraction was suspended with ice-cold 50 mM Tris-HCl (pH 8.1) containing 0.85% NaCl (1 mL), and the suspension was centrifuged as described previously. The washing procedure was repeated, and the pellet fraction was resuspended in a minimum volume of 50 mM Tris-HCl (pH 8.1) containing 0.85% NaCl. An equal volume of 2× SDS-sample buffer was added to the suspension to prepare the samples for western blotting analysis.

### 2.6. Western Blotting Analysis

The protein samples were separated by gel electrophoresis and transferred onto an Immobilon-P polyvinylidene difluoride membrane (Millipore) as previously described [14]. After blocking, the membrane was incubated overnight at 4°C with primary antibodies and then incubated with the biotinylated rabbit anti-mouse IgG (1 : 2000 dilution) or anti-rabbit Ig biotinylated species-specific antibody (1 : 2000 dilution) for 2 h at room temperature. This step was followed by incubation with a streptavidin-horseradish peroxidase conjugate (1 : 500 dilution) for 40 min at room temperature. The western blots were visualized by an enhanced chemiluminescence detection procedure using LAS-1000 (GE Healthcare) image analyzers.

### 2.7. Other Analytical Methods

SDS-PAGE was carried out according to the Laemmli method [15]. Protein concentrations were determined using an advanced protein assay reagent (cytoskeleton) with bovine serum albumin as a standard. The concentrations of the pp2 and pp4 peptides were determined by measuring the Pi released after alkali hydrolysis as previously described [11]. The concentration of the pp6 peptide was determined by measuring the Pi released after acid hydrolysis as previously described [11]. The concentration of the pp10 peptide was determined spectrophotometrically using the absorption coefficient for tyrosine (*ε*_278_ = 1.16 × 10^3^ M^−1^ cm^−1^). 

## 3. Results

### 3.1. Preparation of hCaMKP-N and hCaMKP-N(1–559) Using a Wheat-Embryo Cell-Free Protein Expression System. 

**Figure 2:**
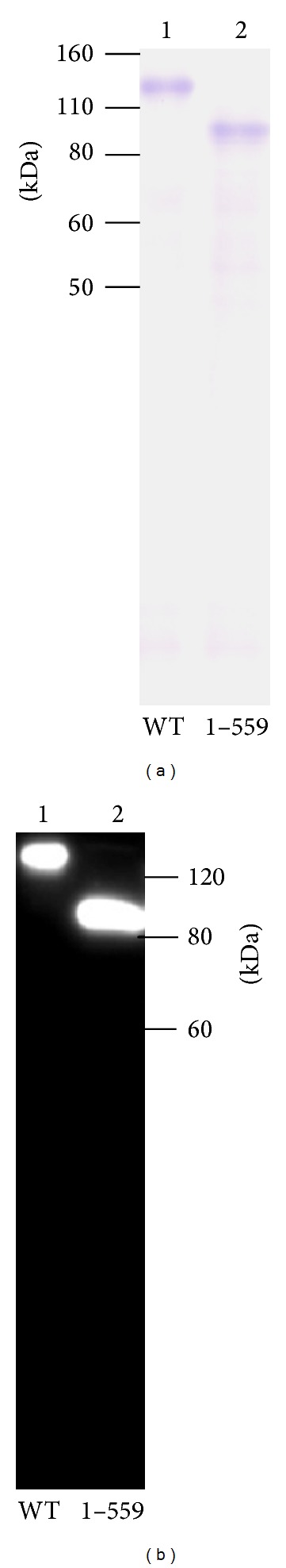
hCaMKP-N and its truncation mutant prepared by a wheat-embryo cell-free protein expression system. (a) hCaMKP-N(WT) (lane 1, 2.5 *μ*g) and hCaMKP-N(1–559) (lane 2, 2.5 *μ*g), which had been prepared and purified as described in Section 2, were subjected to SDS-PAGE on a 10% polyacrylamide gel. The gel was stained with Coomassie Brilliant Blue (CBB) using Quick CBB. (b) hCaMKP-N(WT) (lane 1) and hCaMKP-N(1–559) (lane 2) were subjected to SDS-PAGE on a 10% polyacrylamide gel and analyzed by western blotting analysis using an anti-CaMKP-N antibody (1 : 250 dilution) as the primary antibody.

It has been reported that CaMKP-N undergoes proteolytic processing in the rat brain to generate a 90 kDa fragment in which the C-terminal region is truncated [10]. In an attempt to evaluate the physiological significance of the C-terminal truncation of hCaMKP-N, we prepared full-length and truncated hCaMKP-N to compare their enzymatic properties. Kitani et al. [10] reported that rat CaMKP-N is truncated at or near Pro554. Since the amino acid sequence identity between rat and human CaMKP-N is very high (88%) [16], we constructed expression plasmids for the full-length hCaMKP-N (hCaMKP-N(WT)) and for the truncated hCaMKP-N fragment, in which the C-terminal side of the corresponding Pro residue (Pro559) was deleted, (hCaMKP-N(1–559)), and both contain an added 6× His-tag at their N-terminus (Figure 1(b)). Using the expression plasmids as templates, we synthesized the full-length hCaMKP-N and hCaMKP-N(1–559) proteins with a wheat-embryo cell-free protein expression system and purified these enzymes by Ni^2+^-Sepharose affinity chromatography as described in Section 2. The purified hCaMKP-N(WT) and hCaMKP-N(1–559) showed apparent molecular masses of approximately 120 kDa and 90 kDa on SDS-PAGE, respectively (Figure 2(a)), which are in good agreement with the previous report [5]. Both enzymes were detected by the anti-CaMKP-N antibody that was raised against a synthetic peptide corresponding to the 474–488 region of zCaMKP-N (Figure 2(b)). Because this region shares fairly high homology with the region that is N-terminal side to Pro559 on hCaMKP-N (Figure 1(b)) [8], it was confirmed that the purified phosphatases retained the N-terminal side of Pro559. It should be noted that the hCaMKP-N(WT) preparation was essentially free from the 90 kDa proteolytic fragment usually seen in the preparations purified from the baculovirus-transfected Sf9 cells [5]. 

### 3.2. Activation of hCaMKP-N by C-Terminal Truncation

**Figure 3:**
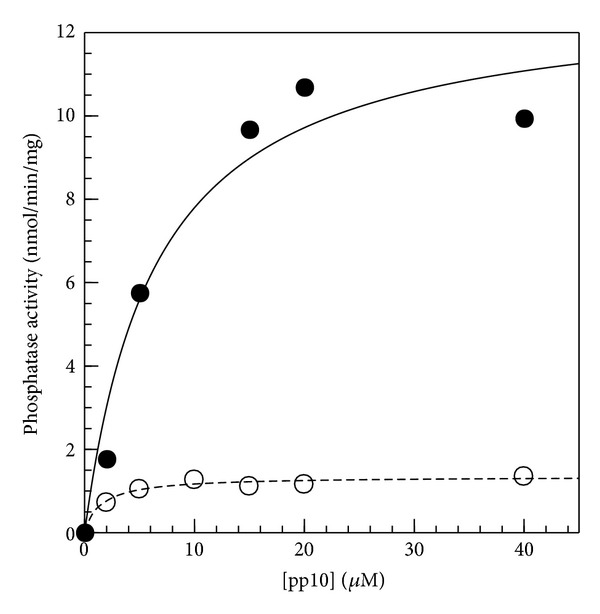
Dephosphorylation of a phosphopeptide substrate pp10 by hCaMKP-N(WT) and hCaMKP-N(1–559). The indicated concentrations of pp10 were dephosphorylated by hCaMKP-N(WT) (open circles) and by hCaMKP-N(1–559) (closed circles) at 30°C as described in Section 2. The amount of inorganic phosphate released in the reaction mixture during the reaction was determined as described. The indicated curves were obtained by direct fits of the data to Michaelis-Menten equation. The data shown are those in a representative experiment of at least three independent determinations with similar results.

Using the protein preparations described previously, the kinetic properties of hCaMKP-N(WT) and hCaMKP-N(1–559) were evaluated and compared. The phosphatase activities were assessed under the standard assay conditions for CaMKP and CaMKP-N [5, 11], where they were assayed in the presence of 2 mM Mn^2+^ using the pp10 phosphopeptide as a substrate (Figure 3). hCaMKP-N(1–559) showed much higher activity than hCaMKP-N(WT). We performed a kinetic analysis of hCaMKP-N(WT) and hCaMKP-N(1–559) with varying concentrations of the pp10 substrate. They displayed typical Michaelis-Menten kinetics, and the *K*_*m*_ value for pp10 and the *V*_max⁡_ value were determined (Figure 3, Table 1). These parameters strongly suggested that the truncation of the C-terminal region of hCaMKP-N results in a marked increase in the *V*_max⁡_ for the phosphatase activity.

### 3.3. Some Enzymatic Properties of hCaMKP-N(1–559)

**Figure 4:**
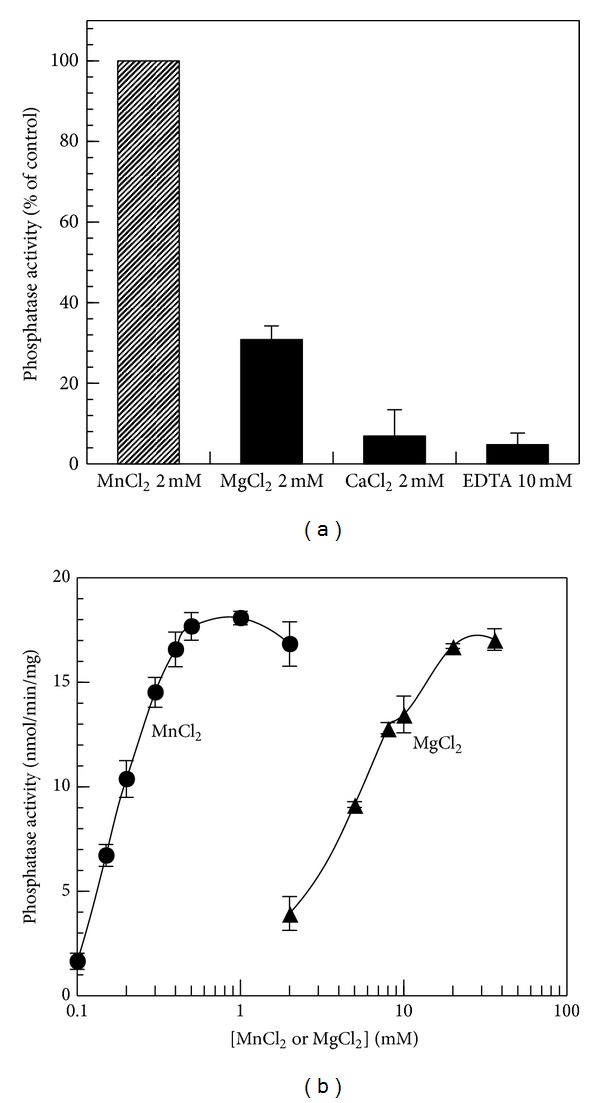
The divalent cation requirement for the phosphatase activity of hCaMKP-N(1–559). (a) hCaMKP-N(1–559) was assayed using pp10 as the substrate in the presence of the indicated divalent cations or EDTA instead of 2 mM MnCl_2_ as described in Section 2. The results are expressed as the percentage of the activity determined with 2 mM MnCl_2_. The data represent the average of three independent experiments ± S.D. (b) hCaMKP-N(1–559) was assayed using pp10 as the substrate in the presence of varying concentrations of MnCl_2_ (circles) or MgCl_2_ (triangles) instead of 2 mM MnCl_2 _.

Since the C-terminally truncated form has been reported to be the most abundant species of CaMKP-N in the rat brain [10], the enzymatic properties of hCaMKP-N(1–559) were further examined using pp10 as the substrate. As shown in Figure 4(a), the truncated fragment showed a strong Mn^2+^-dependent phosphatase activity and a weak Mg^2+^-dependent phosphatase activity but no Ca^2+^-dependent activity. We also examined the effect of varying concentrations of Mn^2+^and Mg^2+^ on the phosphatase activity (Figure 4(b)). Only a submillimolar concentration of Mn^2+^ was required for the full activity of hCaMKP-N(1–559), whereas more than 10 mM Mg^2+^ was required for the same level of activity. A kinetic analysis revealed that the half-maximal activations for Mn^2+^ and Mg^2+^ are 0. 22 ± 0.04 mM and 6.4 ± 1.6 mM, respectively. It should be noted that almost the same level of full activity was observed at their saturating levels, regardless of whether Mn^2+^ or Mg^2+^ was used as a cofactor. 

**Figure 5:**
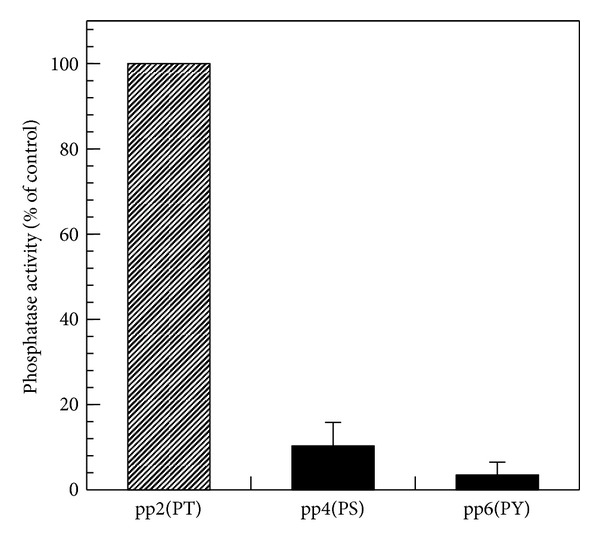
The residue preference at the dephosphorylation site. hCaMKP-N(1–559) was assayed using the indicated phosphopeptides (20 *μ*M) as substrates under the standard assay conditions as described in Section 2. The results are expressed as a percentage of the activity determined using pp2 as the substrate. The data represent the average of three independent experiments ± S.D.

 It has been reported that PPM family phosphatases, including PP2C and CaMKP, strongly prefer phospho-Thr over phospho-Ser as the residue to be dephosphorylated [11, 17]. Therefore, we examined the phosphoamino acid residue preference of hCaMKP-N(1–559). For this purpose, a synthetic phospho-CaMKII(281–289) peptide (named pp2) and its analogs, in which phospho-Thr was replaced with phospho-Ser (named pp4) or with phospho-Tyr (named pp6), were used as substrates for hCaMKP-N(1–559). The truncated hCaMKP-N efficiently dephosphorylated the parent phospho-Thr peptide pp2, but it barely dephosphorylated the phospho-Ser peptide (pp4) and the phospho-Tyr peptide (pp6), indicating strong preference for phospho-Thr residues as was observed for other PPM family phosphatases (Figure 5). 

**Figure 6:**
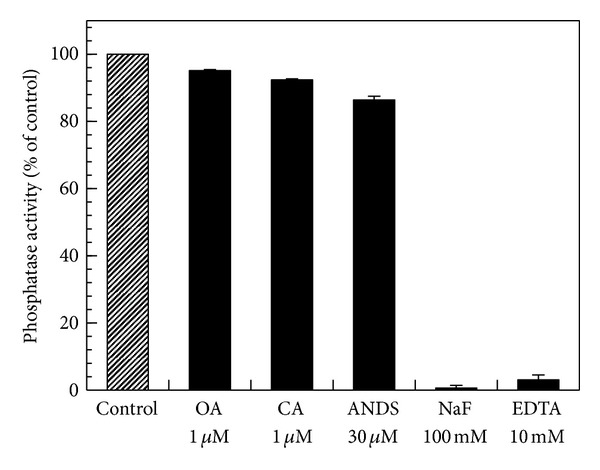
The effects of various inhibitors on the phosphatase activity of hCaMKP-N(1–559). hCaMKP-N(1–559) was assayed using pp10 as the substrate in the presence of the indicated compounds. The results are expressed as a percentage of the activity determined with no compound added (control). The abbreviations used in the figure are as follows: OA: okadaic acid; CA: calyculin A; ANDS: 1-amino-8-naphthol-2,4-disulfonic acid. The data represent the average of three independent experiments ± S.D.

 The effects of some protein phosphatase inhibitors on hCaMKP-N(1–559) were also examined and are shown in Figure 6. Okadaic acid (1 *μ*M) and calyculin A (1 *μ*M), potent PP1 and PP2A inhibitors, had no effect on the phosphatase activity. This is because hCaMKP-N is classified as PPM family phosphatases of which structures are quite different from those of PPP family phosphatases such as PP1 and PP2A. In contrast, NaF (100 mM) and EDTA (10 mM) severely inhibited the phosphatase. It is interesting that ANDS, a CaMKP-specific inhibitor [18], did not inhibit the phosphatase activity of hCaMKP-N(1–559) even at 30 *μ*M, a concentration at which the rat CaMKP was strongly inhibited.

### 3.4. Dephosphorylation of the Autophosphorylated CaMKII in PSD by hCaMKP-N(1–559). 

**Figure 7:**
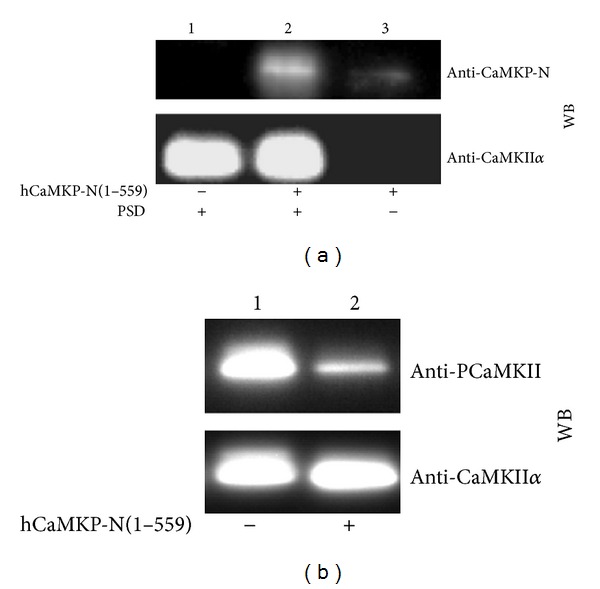
The binding of hCaMKP-N(1–559) to PSD and the dephosphorylation of the autophosphorylated CaMKII. (a) hCaMKP-N(1–559) (1 *μ*g) was incubated on ice for 1 h with (lane 2) or without (lane 3) the PSD fraction (1 *μ*g) as described in Section 2. After incubation, the mixture was centrifuged, and the pellet fraction was washed twice with 50 mM Tris-HCl (pH 7.5) containing 0.85% NaCl, followed by western blotting analysis using anti-CaMKP-N antibody (upper panel). Recovery of the PSD fraction was confirmed by probing the same blot using anti-CaMKII*α* antibody (lower panel). To check the endogenous CaMKP-N levels, the PSD fraction was also incubated in the absence of hCaMKP-N(1–559) as a control (lane 1). (b) The PSD fraction (29 *μ*g/mL), in which CaMKII had been autophosphorylated as described, was incubated at 30°C with (lane 2) or without (lane 1) hCaMKP-N(1–559). After incubation for 30 min, the phosphatase reaction was terminated by adding excess EDTA (20 mM), and aliquots were analyzed by western blotting to examine the extent of phosphorylation at Thr286 on CaMKII (upper panel, anti-PCaMKII) and the total amount of CaMKII*α* on the blot (lower panel, anti-CaMKII*α*). The data presented are representative of at least three independent experiments with similar results.

CaMKII, one of the candidates for the physiological substrate of hCaMKP-N(1–559), is known to exist abundantly in PSD. Therefore, we examined interaction of hCaMKP-N(1–559) and the rat brain PSD as described in Section 2. The endogenous rat CaMKP-N fragment was not detected in the isolated PSD fraction prepared from the rat brain (Figure 7(a), lane 1). Sequence homology between the rat and human CaMKP-N and the sequence homology between the rat and human CaMKII*α* are very high (88% and 99% identity, resp.). After the PSD fraction was incubated with hCaMKP-N(1–559) on ice for 1 h, a significant amount of the CaMKP-N fragment was detected in the PSD fraction (Figure 7(a), lane 2).

 Since the PSD-associated CaMKP-N fragment may play a physiological role, the phosphatase activity of hCaMKP-N(1–559) on the autophosphorylated CaMKII was examined. The endogenous CaMKII in the rat PSD fraction was autophosphorylated in the presence of Ca^2+^/calmodulin and was used as a substrate for hCaMKP-N(1–559). Under the autophosphorylation conditions used, no significant band shift for the CaMKII*α* subunit was observed by SDS-PAGE. As shown in Figure 7(b), the autophosphorylated CaMKII was dephosphorylated by hCaMKP-N(1–559) (lane 2). Therefore, hCaMKP-N(1–559) can bind to PSD to dephosphorylate the autophosphorylated CaMKII. 

### 3.5. The Reversible Regulation of hCaMKP-N(1–559) by Oxidation/Reduction

**Figure 8:**
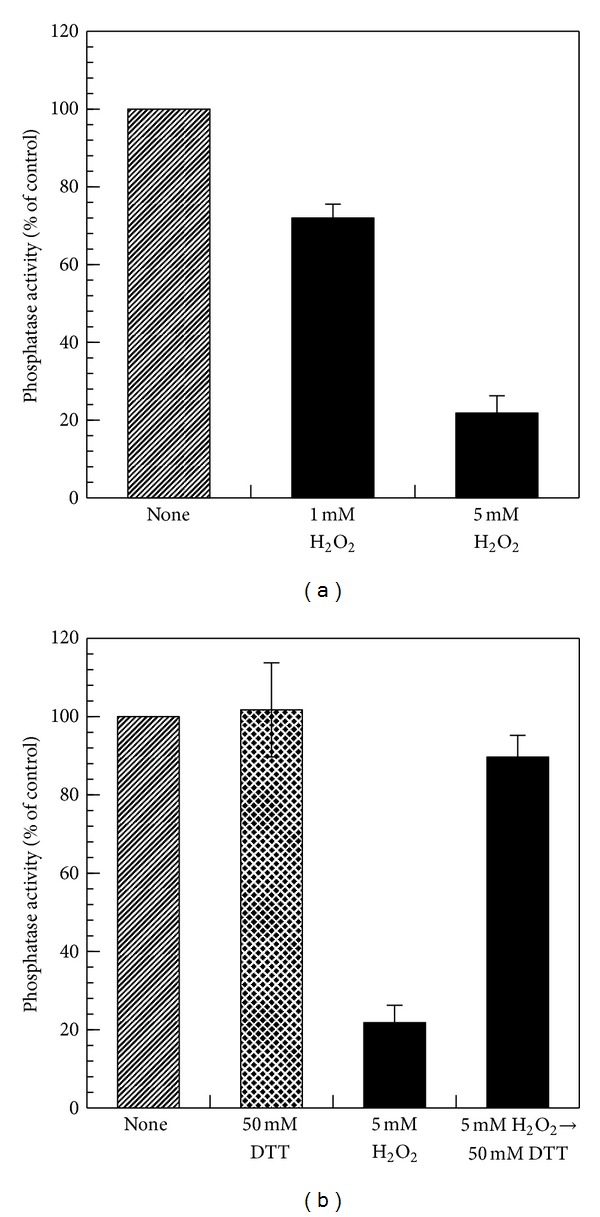
The reversible inactivation of hCaMKP-N(1–559) by H_2_O_2_. (a) hCaMKP-N(1–559) (245 *μ*g/mL) was incubated on ice for 30 min in 25 mM Tris-HCl (pH 7.5) with or without the indicated concentrations of H_2_O_2_, and then the phosphatase activities were determined using pp10 as the substrate. (b) hCaMKP-N(1–559) (245 *μ*g/mL) was incubated on ice for 30 min in 25 mM Tris-HCl (pH 7.5) with 5 mM H_2_O_2_, and then DTT (50 mM) was added for incubation on ice for an additional 30 min. Thereafter, the phosphatase activity was determined using pp10 as the substrate. As a comparison, the phosphatase activity without DTT treatment is also presented (5 mM H_2_O_2_). The DTT treatment itself had no significant effects on the activity of hCaMKP-N(1–559) in the control (50 mM DTT). The results are expressed as a percentage of the control activity (none), which was determined after incubation without any compounds (H_2_O_2_ or DTT) added. The data represent the average of three independent experiments ± S.D.

We have reported that incubation of human CaMKP with H_2_O_2_ leads to the formation of a disulfide bond, which results in inactivation of the enzyme [19]. As shown in Figure 8(a), H_2_O_2_ also inactivated hCaMKP-N(1–559) in a dose-dependent manner. When the inactivated hCaMKP-N(1–559) was further incubated on ice for 30 min with the reducing agent DTT, the phosphatase activity was restored to almost original levels (Figure 8(b)). This indicates that the inactivation of hCaMKP-N(1–559) by H_2_O_2_ is a reversible process and that hCaMKP-N(1–559) is reversibly regulated by oxidation/reduction.

## 4. Discussion

Based on the subcellular localization of transiently expressed hCaMKP-N in COS cells [5], it had been assumed that mammalian CaMKP-N is localized only in the nucleus. However, Kitani et al. [10] showed that in the rat brain, CaMKP-N undergoes proteolytic processing to form a 90 kDa fragment that is localized mainly in the cytosol. Similar proteolytic fragment of CaMKP-N was also found in human frontal cortex [20]. Therefore, the truncated form of hCaMKP-N may have important functions in cells. 

 In this study, we used a wheat-embryo cell-free protein expression system for preparation of hCaMKP-N and its fragment to minimize proteolysis during the expression and purification of hCaMKP-N. Using this system in conjunction with conventional Ni^2+^-NTA agarose chromatography, we were able to individually prepare the full-length hCaMKP-N, hCaMKP-N(WT), and its proteolytic fragment, hCaMKP-N(1–559), for the first time without mutual contamination. Typically, approximately 15 *μ*g of purified hCaMKP-N or its fragment could be obtained from 1 well of a translation reaction mixture (21 *μ*L of the wheat-embryo translation mixture and 206 *μ*L of the substrate solution) in a 96-well microtiter plate (data not shown). The wheat-embryo cell-free protein expression system has been proven useful for the preparation of the proteins that are readily degraded by cellular proteases. Because the protease activities detected in the cell-free system are very low [21, 22], this method may be applicable to other protease-sensitive proteins that are difficult to prepare using conventional cell-based expression systems such as Sf9 cells. 

 Using these preparations, we could rigorously compare the catalytic properties of hCaMKP-N(1–559) and hCaMKP-N(WT). Both hCaMKP-N species have phosphatase activities in the presence of Mn^2+^ toward the pp10 phosphopeptide substrate, which was based on the amino acid sequence around the critical Thr286 autophosphorylation site on CaMKII. This result indicates that the proteolytic fragment is not a degraded and inactive species, but instead, it has phosphatase activity. Although the *K*_*m*_ value for the fragment was somewhat higher than the *K*_*m*_ value for the hCaMKP-N(WT), the *V*_max⁡_ value for the fragment was more than ten times higher than that for the WT. Therefore, we suggest that the truncation of C-terminal region 560–757 of hCaMKP-N is a post-translational regulatory mechanism to generate a highly active species. 

 The mechanism of activation by truncation remains unclear. The truncated C-terminal region might act as an autoinhibitory domain, as is the case for calcineurin [23]. Alternatively, processing of the region might cause a conformational change in its catalytic center that leads to catalytic activation. It has been reported that some protein phosphatases in the PPP family are activated by proteolysis [24, 25]. We have also reported that zCaMKP-N is activated by proteolytic processing of the C-terminal domain [9]. Therefore, activation by C-terminal truncation appears to be a common feature for CaMKP-N, despite the fact that hCaMKP-N and zCaMKP-N have fairly different molecular sizes and primary structures. Because it has been reported that rat CaMKP-N(1–554), a fragment corresponding to hCaMKP-N(1–559), is localized in the cytosol of transfected COS cells [10], the truncation of the C-terminal domain is likely to regulate catalytic activity as well as the intracellular localization of hCaMKP-N. Since inhibition of the proteolytic processing of zCaMKP-N in Neuro2a cells by proteasome inhibitors significantly changed substrate targeting in the cells [9], activation and translocation of the mammalian CaMKP-N may also affect the intracellular substrate targeting. 

 The activated CaMKP-N fragment generated by the proteolytic processing is reported to be the major species of CaMKP-N in the rat brain [10]. Here, we show a molecular characterization of the 90 kDa active fragment in human, hCaMKP-N(1–559). It exhibited okadaic acid/calyculin A-insensitive and Mn^2+^ or Mg^2+^-dependent phosphatase activity and demonstrated a striking preference for a phosphothreonyl peptide over a phosphoseryl or a phosphotyrosyl peptide. These enzymatic properties are similar to those of CaMKP [11]. However, the metal dependence of hCaMKP(1–559) was somewhat different from that of CaMKP. Although the half-maximal activation for Mn^2+^ is comparable to that of rat CaMKP (~0.2 mM), activation by Mg^2+^ is more prominent in hCaMKP(1–559) than it is in CaMKP [26]. Furthermore, hCaMKP-N(1–559) showed Mn^2+^-dependent activity and comparable Mg^2+^-dependent activity at its saturating levels. NaF is known to inhibit various protein phosphatases. Fluoride is reported to directly bind to the metal ions in the active center of bovine purple acid phosphatase [27]. Since Ser/Thr protein phosphatases are known to be metalloenzymes that employ dinuclear metal center similar to purple acid phosphatases [28], it is most likely that fluoride also binds to the metal center to inhibit its phosphatase activity. Interestingly, ANDS, a potent inhibitor for CaMKP [18], did not inhibit the phosphatase activity of hCaMKP-N(1–559). This suggests that the three-dimensional structure of the active site of hCaMKP-N is considerably different from that of rat CaMKP even though their primary structures of their putative catalytic regions are highly homologous. 

 hCaMKP-N(1–559) could bind to PSD to dephosphorylate the CaMKII associated with it. Based on electron microscope results, the corresponding CaMKP-N fragment is suggested to be concentrated in PSD together with CaMKII in rat brain [10]; however, the endogenous rat CaMKP-N fragment was not detected in the isolated PSD fraction. Therefore, it is likely that the CaMKP-N fragment is not a component of PSD, but its binding to PSD is dynamically regulated in neuronal cells. This fragment might be involved in regulation of CaMKII activity in PSD, where synaptic transmission is tightly controlled. The CaMKP-N activated by proteolysis might be a critical regulator for synaptic transmission through controlling the phosphorylation state of the CaMKII in PSD.

 Another notable finding in this study is that hCaMKP-N(1–559) is inactivated by H_2_O_2_ treatment, and reactivated by incubation with DTT. Recently, we reported that human CaMKP is reversibly regulated by oxidation/reduction at Cys359 [19]. This Cys residue is adjacent to an Asp residue that is essential for metal binding at the active site, and the Cys-Asp sequence is conserved in the catalytic sites of many PPM family enzymes including hCaMKP-N. Therefore, the observed inactivation of hCaMKP-N(1–559) might be due to reversible oxidation at Cys436 of hCaMKP-N. The reversible regulation of the phosphatase activity of hCaMKP-N(1–559) by oxidation/reduction may be an important mechanism for regulating the phosphorylation levels of CaMKII in neuronal cells, especially in PSD, in response to oxidative stress.

 In summary, we showed for the first time that hCaMKP-N is activated through truncation of the C-terminal domain. The active truncated fragment could bind to PSD to dephosphorylate CaMKII, and its enzymatic properties were similar to those of CaMKP except for its Mg^2+^-dependence and sensitivity to ANDS. Very recently, genome-wide association studies suggested that single nucleotide polymorphisms found in the loci for CaMKP-N (PPM1E) and for CaMKP (PPM1F) are associated with testicular germ cell tumor [29] and with both schizophrenia and bipolar disorders [30], respectively. Since hCaMKP-N(1–559) is supposedly localized in the cytosol where CaMKP is found, the next important question concerns what are the roles that CaMKP and the active CaMKP-N fragment share in the cytosol. Further work is needed to address this question. 

## Figures and Tables

**Table 1:** Comparison of the phosphatase activities of hCaMKP-N(WT) and hCaMKP-N(1–559). hCaMKP-N(WT) and hCaMKP-N(1–559) were assayed using varying concentrations of the pp10 substrate as described in Section 2. Michaelis-Menten kinetic parameters were determined from a direct fit to the Michaelis-Menten equation. The data represent the average of three independent experiments ± S.D.

	*K*_*m*_ (*μ*M)	*V*_max⁡_ (nmol/min/mg)
hCaMKP-N(WT)	2.0 ± 0.9	1.4 ± 0.1
hCaMKP-N(1–559)	5.4 ± 0.1	16.3 ± 3.6

## Abbreviations

ANDS: 1-Amino-8-naphthol-2,4-disulfonic acid
CaMKII: Ca^2+^/calmodulin-dependent protein kinase II
CaMKP: Ca^2+^/calmodulin-dependent protein kinase phosphatase
hCaMKP-N: Human Ca^2+^/calmodulin-dependent protein kinase phosphatase-N
PSD: Postsynaptic density
zCaMKP-N: Zebrafish Ca^2+^/calmodulin-dependent protein kinase phosphatase-N.
